# A new genus of protorhyssaline wasps in Raritan amber (Hymenoptera, Braconidae)

**DOI:** 10.3897/zookeys.711.20709

**Published:** 2017-10-23

**Authors:** Michael S. Engel, Jennifer C. Thomas, Abdulaziz S. Alqarni

**Affiliations:** 1 Division of Entomology, Natural History Museum, 1501 Crestline Drive – Suite 140, University of Kansas, Lawrence, Kansas 66045-4415, USA; 2 Department of Ecology & Evolutionary Biology, University of Kansas, Lawrence, Kansas 66045, USA; 3 Division of Invertebrate Zoology, American Museum of Natural History, Central Park West at 79th Street, New York, New York 10024-5192, USA; 4 Department of Plant Protection, College of Food and Agriculture Sciences, King Saud University, P.O. Box 2460, Riyadh 11451, Kingdom of Saudi Arabia

**Keywords:** Apocrita, Ichneumonoidea, parasitoid, Turonian, taxonomy

## Abstract

A second species of protorhyssaline wasps (Braconidae) is described and figured from inclusions in Upper Cretaceous (Turonian) amber of the Raritan Formation in New Jersey, USA. *Rhetinorhyssalites emersoni*, **gen. n., sp. n.**, is distinguished from other protorhyssalines, particularly the contemporaneous *Protorhyssalus goldmani*.

## Introduction

The parasitoid wasp subfamily includes a variety of generally plesiomorphic cyclostome braconids known only from Cretaceous deposits. The subfamily was initially described based on a species from the Upper Cretaceous (Turonian) amber of New Jersey (Basibuyuk et al. 1999), but was subsequently recognized in Lower Cretaceous amber from Spain (Ortega-Blanco et al. 2011) as well as Upper Cretaceous amber from Myanmar, France, and Canada (Perrichot et al. 2009, Engel and Wang 2016, Engel 2016), bringing the total to seven species and genera. These wasps are generally plesiomorphic in most traits relative to other cyclostome lineages and the few attempts to place them within a phylogenetic framework with other basal Braconidae failed to recover the subfamily as monophyletic, the two genera then known falling into an extensive basal polytomy (Perrichot et al. 2009). In fact, the lack of defining apomorphies was noted by the original authors when proposing the subfamily (Basibuyuk et al. 1999), and as more specimens and taxa become known future analyses may demonstrate protorhyssalines to be a grade, necessitating their breakup. Nonetheless, despite the rarity of Braconidae in Cretaceous resins, most of the known species can be ascribed to this assemblage of plesiomorphically similar and generalized braconids (e.g., Basibuyuk et al. 1999, Perrichot et al. 2009, Ortega-Blanco et al. 2011, Quicke 2015, Engel and Wang 2016).

**Figures 1–3.**
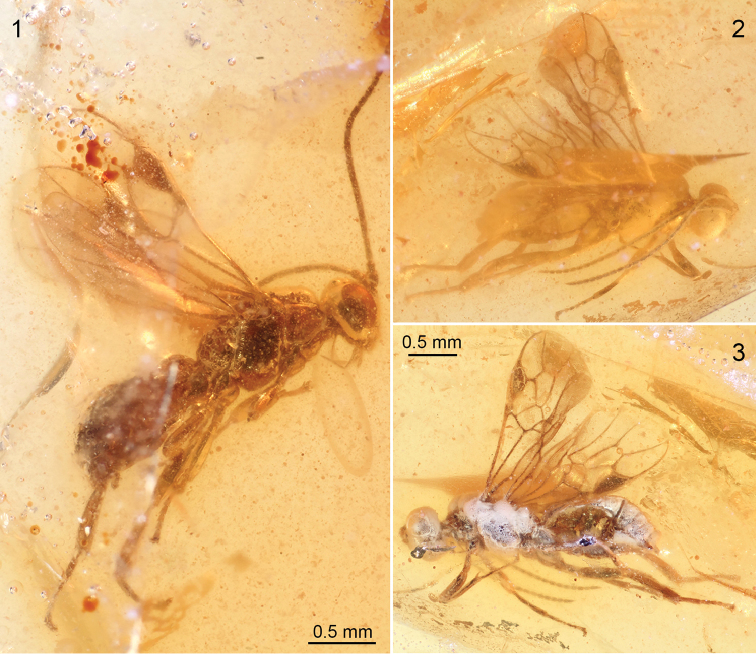
Photographs of males of *Rhetinorhyssalites emersoni*, gen. et sp. n., in Raritan amber. **1** Right lateral habitus of holotype (AMNH NJ-892A) **2** Right lateral view of paratype (AMNH NJ-692), to same scale as figure C **3** Left lateral view of paratype.

Here we describe a second species of protorhyssaline wasps (Fig. 1) from the same Upper Cretaceous (Turonian) deposits as the type genus of the subfamily. The new species has some similarities with a slightly older species from the Cenomanian of Myanmar, and is placed in a genus distinct from other protorhyssalines based particularly on features of the wing venation.

## Material and methods

Two individuals were identified in slightly turbid amber pieces from the Upper Cretaceous Raritan Formation of New Jersey. The amber has been dated palynologically to the Turonian, at approximately 90 Ma, and the localities mapped by Grimaldi et al. (2000) and Grimaldi and Nascimbene (2010). The pieces were embedded in epoxy following the procedure of Nascimbene and Silverstein (2000) and the surfaces polished flat, giving lateral views of the inclusions. The holotype is complete but positioned near edges of the amber preventing direct facial and dorsal views (Fig. 1), and the animal’s left side is partially obscured by adjoining bubbles. The paratype is covered in places by a fine layer of fine, microscopic bubbles and the metasoma is damaged, opened laterally and infilled by amber, but otherwise complete (Figs 2, 3). Both pieces are deposited in the American Museum of Natural History, New York.

The descriptions are formatted like those recently presented for related Cretaceous braconids (e.g., Engel and Wang 2016, Engel 2016), with morphological terminology generally based on Huber and Sharkey (1993) and Sharkey and Wharton (1997). Microphotographs were taken with the aid of an Infinity K-2 lens and Canon 7D digital camera, while measurements and the wing drawings were made using ocular micrometers and a camera lucida, respectively, and affixed to an Olympus SZX-12 stereomicroscope. Measurements of the holotype are provided with those of the paratype, when possible, in parentheses.

## Systematic paleontology

### Family Braconidae Nees von Esenbeck

#### Subfamily

Taxon classificationAnimaliaHymenopteraBraconidae

Basibuyuk et al.

##### Included genera.

*Archaeorhyssalus* Engel in Engel & Wang (2016), *Diorhyssalus* Engel (2016), *Protorhyssalodes* Perrichot et al. (2009), *Protorhyssalopsis* Ortega-Blanco et al. (2011), *Protorhyssalus* Basibuyuk & Quicke in Basibuyuk et al. (1999), *Rhetinorhyssalites* gen. n. (*vide infra*), and *Rhetinorhyssalus* Engel (2016).

#### Rhetinorhyssalites gen. n.

Taxon classificationAnimaliaHymenopteraBraconidae

http://zoobank.org/E6F92137-F91C-479C-8B8D-C0055517E109

##### Type species.

*Rhetinorhyssalites emersoni* sp. n.

##### Diagnosis.

**Figure 4.**
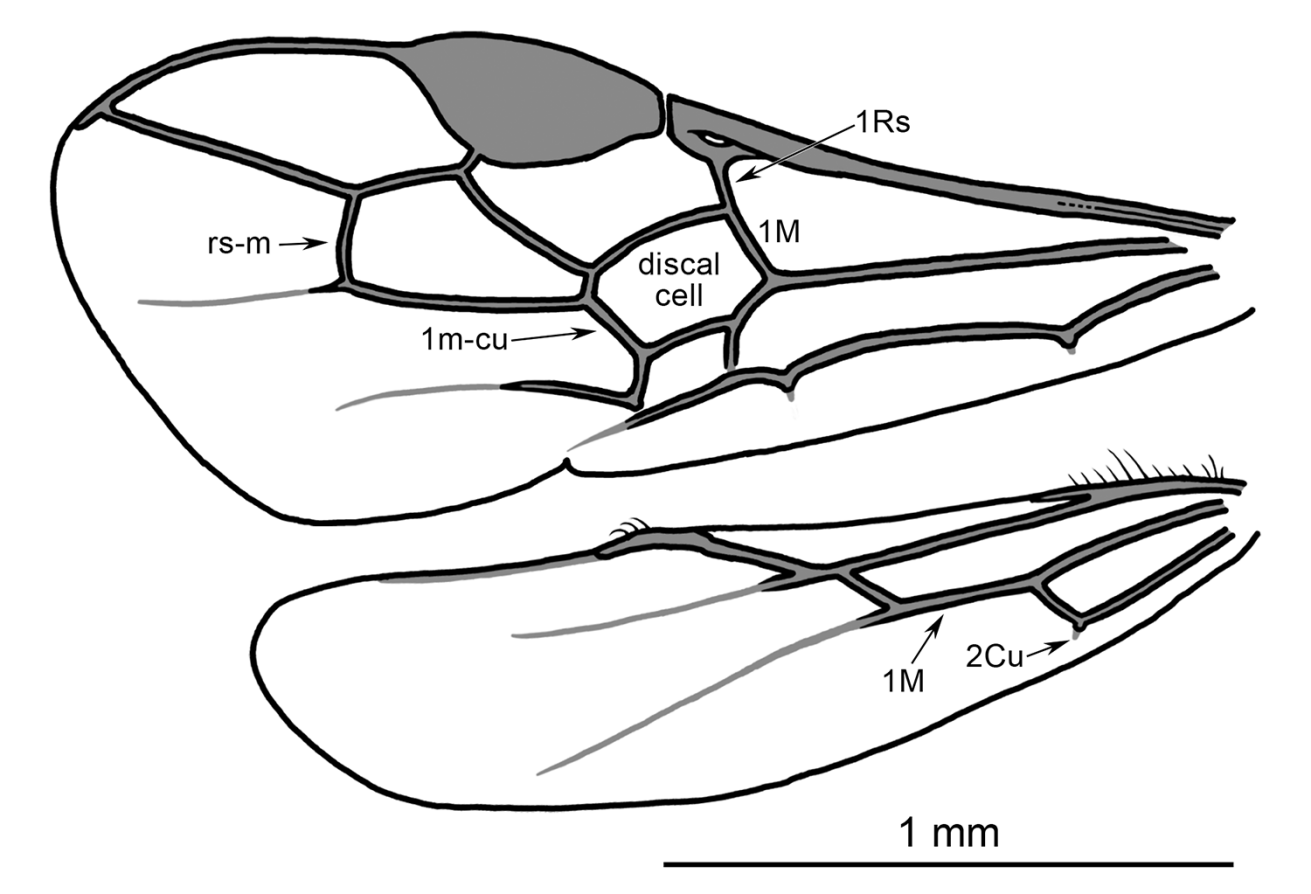
Wing venation of *Rhetinorhyssalites emersoni*, gen. et sp. n., with most marginal setae omitted; forewing above, hind wing below.

Head cyclostome, with hypoclypeal depression deep; antenna with 20–24 flagellomeres (18–20 in *Protorhyssalus* Basibuyuk et al.); flagellum with scattered multiporous plate sensilla; occipital carina present and complete, albeit particularly weak dorsally; compound eyes not emarginate, without evident setae. Pronotal collar short, with subpronope scarcely indicated; notauli deeply impressed, percurrent; mesoscutal lateral areas sculptured as on remainder of mesoscutum; mesoscutellum not raised relative to mesoscutum (distinctly raised in *Protorhyssalus*); epicnemial carina absent (present in *Protorhyssalus*: “prepectal carina” sensu Basibuyuk et al. 1999); postpectal carina absent. Forewing (Fig. 4) with minute costal cell apically, otherwise C+Sc+R fused, without indication of fusion line except proximally; 1Rs present, forming straight line with 1M (1Rs/1M straight), slightly more than one-half length 1M (very short in *Protorhyssalus*); rs-m present; 1m-cu meeting second submarginal cell, second submarginal cell narrowly elongate postero-proximally (not so in *Protorhyssalus*); 2m-cu absent; 1cu-a strongly postfurcal; 2cu-a scarcely present (represented only by hint of stub at angle in 3Cu; stubs 1a and 2a present. Hind wing with sc+r-m lacking bulla, much shorter than 1M; bulla lacking between 1A and stub of 2Cu (present in *Protorhyssalus*); minute stub of 2Cu present. Metasomal tergum I with dorsope and laterope deeply impressed; dorsal carina strong, extending to posterior margin of tergum.

##### Etymology.

The generic name is a combination of *Rhetinorhyssalus* Engel, a genus with somewhat similar venation, and the suffix –*ites* (Greek, “having the nature of”). The gender of the name is feminine.

#### Rhetinorhyssalites emersoni sp. n.

Taxon classificationAnimaliaHymenopteraBraconidae

http://zoobank.org/CDEBECC1-2E8A-412C-88E5-689DCD502431

Figs 1–3 , 4

##### Holotype.

♂, AMNH NJ-892A; deposited in the Division of Invertebrate Zoology, American Museum of Natural History, New York.

##### Paratype.

♂, AMNH NJ-692; same locality and repository as the holotype.

##### Locality and horizon.

Upper Cretaceous (Turonian) amber, New Jersey, Middlesex County, Sayreville, white oaks pit. The locality has been discussed and the Raritan amber deposits mapped by Grimaldi et al. (2000) and Grimaldi and Nascimbene (2010).

##### Diagnosis.

As for the genus (*vide supra*).

##### Description.

♂: Total length 2.54 mm as preserved (2.53 mm); forewing length 1.98 mm (1.90 mm), hind wing length 1.66 mm (1.60 mm); integument, where evident, dark brown, lighter on appendages; wing veins dark brown to brown, membranes hyaline and clear.

Head apparently about as long as wide (direct frontal view not possible in either holotype or paratype), with small punctures separated by about 2 or more times a puncture width, integument between smooth, with scattered, suberect, minute setae, setae more numerous on lower face; face below antennal toruli somewhat flat; clypeus slightly protruding, rounded, short; hypoclypeal depression deep and wide; mandible short (mandibles closed in both specimens); labial palpus short, apparently with three palpomeres; maxillary palpus elongate, apparently slightly longer than head, with six palpomeres, palpomeres IV–VI elongate, thinner than preceding palpomeres, palpomere III thickened and dorsally hunched, with abundant distinctive setae dorsally; compound eye large and glabrous, length 0.36 mm, broader than gena, inner margin not emarginate; ocelli positioned close together on top of vertex; occipital carina complete, weak dorsally; antenna slightly shorter than body length; scape squat, only slightly longer than wide, length 0.11 mm, width 0.09 mm, truncate apically; pedicel about as long as wide, slightly narrower than scape, length 0.07 mm, width 0.06 mm; flagellum with 20 flagellomeres (24 flagellomeres); basal flagellomeres elongate, approximately 3–4 times as long as wide, flagellomere I length 0.16 mm, width 0.04 mm; flagellomere II length 0.14 mm, width 0.04 mm; flagellomere III length 0.13 mm, width 0.04 mm; remaining flagellomeres progressively shorter, apical flagellomeres about 1.25–2.0 times as long as wide; multiporous plate sensilla sparse.

Mesosoma length 0.98 mm (0.98 mm); pronotal surface smooth; mesoscutum with minute, setigerous punctures separated by a puncture width or less, integument between punctures smooth; notauli deeply impressed, crenulate, percurrent; lateral sectors of mesoscutum (outside of notauli) distinctly raised, convex, with sculpturing as on remainder of mesoscutum; mesoscutellar sulcus deeply impressed; mesoscutellum not raised, on same level with mesoscutum; mesopleuron largely smooth and impunctate, with borders areolate; sternaulus absent; metapleuron areolate; propodeum coarsely and deeply areolate. Legs slender, with numerous minute setae; tibial spurs short, protibial calcar slightly curved, without comb; metafemur tubular except with weak subapical concavity on inner ventral surface; metatibia length 1.26 mm (1.23 mm); basitarsi longest tarsomeres, but shorter than combined length of remaining tarsomeres, slightly longer than fifth tarsomeres; pretarsal claws short, simple; arolium small. Forewing (Fig. 4) with minute costal cell present apically near pterostima, remainder of C+Sc+R completely fused with faint indication of fusion line proximally; pterostigma large, longer than wide, border inside marginal cell faintly convex, anterior border bulging; marginal cell large, extending nearly to wing apex; R slightly extending beyond marginal cell apex along apical wing margin to wing apex; 1Rs present, slightly more than one-half length 1M; 1Rs/1M straight; Rs+M weakly arched (nearly straight); 1m-cu entering second submarginal cell near base, thus short 2M present (and “2Rs+M” lacking); 2M angled posteriorly, giving second submarginal cell narrowly elongate extension postero-proximally; 2Rs elongate; r-rs arising in apical half of pterostigma, short, shorter than 1Rs; 1rs-m present, about as long as 3Rs; 3M much longer than 2Rs; 1cu-a strongly postfurcal (positioned beyond one-third discal cell length); 1Cu about as long as 1cu-a; 2Cu longer than 1Cu; 2cu-a present only has hint of stub, with subdiscal cell minutely open apically; stubs of 1a and 2a present. Hind wing (Fig. 4) with margins setose; three distal hamuli present on R and set of “secondary hamuli” (sensu Basibuyuk et al. 1999) on proximal portion of C; R tubular for short distance along with margin, otherwise extending as nebulous vein, terminating well prior to wing apex; 2Sc+R extremely short; Rs tubular for short distance then extending as nebulous vein; sc+r-m without bulla, longer than 2Sc+R, much shorter than 1M; 2M tubular near base then nebulous; 1Cu much shorter than 1M; 2Cu present as minute stub; bulla lacking between 1A and 2Cu stub.

Metasoma length 1.21 mm (1.20 mm), with terga II and III fused and with distinct suture line; integument generally smooth an impunctate, with scattered, short, appressed setae; first metasomal tergum with dorsal carinae strong, extending to posterior tergal margin, dorsopes deeply impressed and areolate; lateral carinae strong, with lateropes deeply impressed; tergum I about as long as wide, remaining terga wider than long.

♀: *Latet*.

##### Etymology.

The specific epithet honors the late William K. Emerson (1925–2016), a leading malacologist with the American Museum of Natural History (Mikkelsen and Landman 2017) and good friend to the senior author for the last 20 years of his life. In 1999, after one of many relaxing enjoyable chats and before I (M.S.E.) departed, Bill pulled from a shelf his copy of his 1976 guide to shells (Emerson and Jacobson 1976), autographed it, and placed it in my hands. It remains a treasured possession and reminder of joyful days and Bill’s kindness and good humor.

## Discussion

As the name implies, there is some similarity in the wing venation between *Rhetinorhyssalites emersoni* and the slightly older *Rhetinorhyssalus morticinus* Engel from Cenomanian amber of Myanmar (Engel 2016). Although there are many differences between these two taxa, such as the complete absence of an occipital carina; shortened 1Rs, shorter rs-m relative to 3Rs, longer r-rs, more basad 1cu-a in the forewing; and absence of both 2Cu and a bulla between 1A and 1Cu in the hind wing (Engel 2016), the general appearance of the forewings are superficially similar. In both species the form of the submarginal cell is quite similar, particularly in the narrowly elongate proximal extension of the second submarginal cell (Fig. 4). *Rhetinorhyssalites* differs from the coeval *Protorhyssalus* in Raritan amber (Basibuyuk et al. 1999) in the unraised mesoscutellum (strongly raised in the latter); the absence of an epicnemial carina (present in the latter); forewing 1Rs long, proximally extended second submarginal cell, and more prominently postfurcal 1cu-a; and in the hind wing the scarcely present 2Sc+R, the absence of a bulla in 1A, and the scarcely evident 2Cu stub. The former species is also slightly larger, approximately 2.5 mm in length versus 1.5–2.0 mm in the latter, and has a larger number of flagellomeres (20–24 in *R. emersoni* versus 18–20 in *Protorhyssalus*); however, until both sexes are known for both genera these differences shall require further testing. The more prominent 1Rs and absence of a bulla between 1A and 1Cu is distinctive relative to all other protorhyssalines, although most characters agree more closely with *Protorhyssalus* than any other genera.

Unfortunately, protorhyssalines remain a great rarity and it is impossible at present to elaborate more fully on these early parasitoids, particularly in regards to their biology. Putatively primitive braconids such as rhyssalines, are ectoparasitoids of larval Coleoptera and Lepidoptera (Quicke 2015), and it may be that this biology is plesiomorphic for the clade, and potentially shared symplesiomorphically with protorhyssalines. However, the biology of basal groups such as and remain unknown and this could alter any interpretation of groundplan host associations for Braconidae. Nonetheless, parasitism of wood-boring larva is often primitive within apocritan clades (Grimaldi and Engel 2005), and perhaps this trend is true for Ichneumonoidea and basal Braconidae, with those rhyssalines found on arboreal moth larvae representing isolated shifts away from more typical wood-infesting lineages. The female of *Archaeorhyssalus subsolanus* Engel (Engel and Wang 2016) has a moderately long ovispositor which is at least consistent with possible wood-boring hosts. Regardless, the continued discovery of Cretaceous braconids, particularly those preserved in amber, will hopefully add considerable character data toward resolving basal relationships and host associations within this hyperdiverse family.

The Cretaceous diversity of Braconidae remains scarcely known, although a significant expansion in our knowledge has been made during the last 20 years. Although the family is vast and cosmopolitan today, with about 20,000 described species, its fossil record is scant despite extending well into the Mesozoic. The discovery of *Rhetinorhyssalus emersoni* in Turonian Raritan amber expands not only our general understanding of the faunal composition of Hymenoptera from the Raritan Formation of eastern North America, but builds upon our meager knowledge of Braconidae from the Cretaceous. Although we still look ‘through a glass darkly’, the continued discovery and description of further species such as *R. emersoni* remains our only means of clearing our view into the distant history of the braconids and other significant diversifications.

## Supplementary Material

XML Treatment for Protorhyssalinae

XML Treatment for Rhetinorhyssalites

XML Treatment for Rhetinorhyssalites emersoni
